# A Phase I, Randomized, Double-Blind, Placebo-Controlled Clinical Trial to Evaluate the Safety and Tolerance of Oil Palm Phenolics (OPP) in Healthy Volunteers

**DOI:** 10.3389/fphar.2022.893171

**Published:** 2022-06-20

**Authors:** Nur Balqis Muhammad Ismail Tadj, Nurul ‘Izzah Ibrahim, Qodriyah Haji Mohd Saad, Tg Mohd Ikhwan Tg Abu Bakar Sidik, Soon-Sen Leow, Syed Fairus, Isa Naina Mohamed

**Affiliations:** ^1^ Pharmacoepidemiology and Drug Safety Unit, Department of Pharmacology, Faculty of Medicine, Universiti Kebangsaan Malaysia Medical Centre, Kuala Lumpur, Malaysia; ^2^ Malaysian Palm Oil Board (MPOB), Kajang, Malaysia

**Keywords:** oil palm phenolics, clinical trial (CT), antihyperlipidemia, antioxidant, natural products, safety and toxicology

## Abstract

**Background and aim**: Oil palm aqueous by-products rich in phenolic content are known as oil palm phenolics (OPP), and pre-clinical research has shown that OPP has great potential to be further developed as an anti-hyperlipidemic agent. Hence, in order to introduce OPP into market, its safety profile needs to be established by undergoing a phase I clinical trial on healthy humans.

**Methods**: A parallel, placebo-controlled, randomized, and double-blinded clinical trial was conducted for 2 months on 100 healthy subjects aged 20–40 years old. This trial was registered at clinicaltrials.gov (NCT04164446). The subjects were randomly allocated to four treatment arms with 25 participants each: placebo, 250, 1,000, and 1,500 mg of OPP. During the trial, subjects were required to consume four capsules simultaneously per day. Withdrawal of fasting blood for hematology, liver and renal function analysis, and medical examination were conducted at baseline (day 1), day 30, and day 60. For monitoring, vital signs (blood pressure and pulse rate) and weight measurements were taken during each visit.

**Results**: Minor adverse events (AEs) were reported in all groups especially at high dose (1,500 mg) but none were serious adverse events (SAEs). Fasting blood parameters between control and all OPP-treated groups demonstrated no statistically significant difference from baseline to day 60.

**Conclusion**: With no major AEs and SAEs reported and no abnormal findings in biochemistry and hematology results, OPP supplementation in capsule form is safe to be taken up to 1,500 mg a day.

## 1 Introduction

Being the second largest palm oil producer in the world with the production of 19.86 million tons of crude palm oil in 2019, Malaysia generates a huge amount of biowaste annually (Malaysian Palm Oil Board, 2020). The manufacturing process in the palm oil industry produces both solid waste (i.e., mesocarp fruit fibers, empty fruit bunch, and palm kernel shell) and liquid waste which is the palm oil mill effluent (POME). Both of these wastes contribute negatively to the surrounding environment especially POME (Kamarudin et al., 2015). A novel process to recover water-soluble oil palm phenolics (OPP) from POME has been developed and subsequently may transform a bioburden into a range of health applications (Sambanthamurthi et al., 2008).

OPP has demonstrated significant scavenging activity with a half-life (t^1/2^) of less than 30 s in a 2,2-diphenyl-1-picrylhydrazyl (DPPH) assay. This potent antioxidant activity of OPP might be due to its high phenolic content that is mainly contributed by caffeoylshikimic acid at a concentration of 10,800 ± 2,400 mg/kg followed by p-hydroxybenzoic acid and protocatechuic acid with concentrations of 7,000 ± 1,000 and 600 ± 100 mg/kg, respectively (Sambanthamurthi et al., 2011). The ability to scavenge free radicals and donate hydrogen atoms contributes to the antioxidant activity of OPP, which depends on the degree of hydroxylation of the phenolic compounds (Rice-Evans et al., 1996). The most abundant phenolic acid in OPP, caffeoylshikimic acid, possesses four hydroxyl groups which is responsible for the potent antioxidant activity of OPP. Working together with other phenolic acids in OPP, it might also contribute to the synergistic effect of the antioxidant activity observed. Due to its powerful antioxidant property, OPP was also noted to possess medicinal properties such as anti-tumor (Sekaran et al., 2010), anti-atherogenic (Idris et al., 2014), anti-diabetic (Bolsinger et al., 2014), anti-amyloidogenic (Monplaisir, 2016), and anti-hyperlipidemic properties (Fairus et al., 2018). In terms of toxicity, a 9-weeks acute and 90-days sub-chronic animal toxicity study of OPP showed no observable adverse effect in a human equivalent dose of up to 2,000 mg/kg body weight per day. No significant effects were noted on body weight, food consumption, hematology, clinical chemistry, organ weights, and histopathological examination (Lynch et al., 2017). Therefore, as OPP has various medicinal properties, it is worth conducting a clinical trial in humans in determining its safety and tolerance in healthy subjects. To date, a previous phase I single-blind clinical trial has been conducted to evaluate OPP supplementation in the form of juice in healthy volunteers (Fairus et al., 2018). The dosage used was 450 mg GAE/day, the equivalent to 9,000 mg daily. Both animal and human studies proved that oral OPP consumption is safe, even at high dosage with no risk of causing any abnormality.

In this clinical trial, we incorporated several improvements such as conducting the trial as a randomized double-blinded study, the encapsulated form of OPP, and a much lower dose of OPP for better tolerance and compliance of participants. The aim of this current clinical trial was to evaluate the safety and tolerability of OPP supplementation in encapsulated form among healthy volunteers.

## 2 Materials and Methods

### 2.1 OPP Composition

The major compounds found in OPP extract as patented by Sambanthamurthi et al. (2011) were: caffeoylshikimic acid (C_16_H_16_O_8_), p-hydroxybenzoic acid (C_7_H_6_O_3_), and protocatechuic acid (C_7_H_6_O_4_).

### 2.2 Capsule Composition

Each OPP capsule was composed of different amounts of pure OPP extract; either 62.5, 250, or 375 mg of OPP extract with each participant given four capsules a day to equal the total amount of 250, 1,000, and 1,500 mg, respectively. Meanwhile, the placebo composition was made up of 100% dextrose sugar. To ensure the double-blinded trial was successfully achieved, all the processes involving capsule identification such as encapsulation, packaging, and distribution processes were wholly handled by an appointed contract research organization (CRO).

### 2.3 Inclusion and Exclusion Criteria

Initially, 214 healthy volunteers were recruited from the Klang Valley area between December 2019 and January 2020. Volunteers were recruited at the OPP Booth (located at the HCTM Main Lobby), *via* online advertisements and through posters and flyers. From the 214 volunteers recruited, only 100 volunteers (68 women and 32 men) were selected based on the inclusion and exclusion criteria. The inclusion criteria were as follows: healthy subjects aged 20–40 years, non-smoker, non-alcoholic, did not consume any antioxidant supplements (volunteers were required to stop taking any supplements a month before the trial started), total cholesterol <5.2 mmol/L, LDL cholesterol <3.36 mmol/L, and triglycerides <1.69 mmol/L. The exclusion criteria included the following: current use of lipid-lowering therapies or anti-hypertensive drugs; medical history of cardiovascular disease, diabetes, or dyslipidemia; smoking, habitual alcohol consumption, and pregnant or lactating women. All subjects were advised of the potential side effects of the study medication. Volunteers gave their written informed consent before the beginning of the trial.

### 2.4 Study Design and Protocol

This trial was a mono-centric, parallel, placebo-controlled, randomized, and double-blind study conducted at the clinical trial ward (CTW), Hospital Canselor Tuanku Muhriz (HCTM), Kuala Lumpur and was performed according to Good Clinical Practice (GCP). This clinical trial was approved by the Research Ethics Committee of Universiti Kebangsaan Malaysia (RECUKM) (UKM PPI/111/8/JEP-2019-100) and all the procedures were done according to the Declaration of Helsinki and Malaysian GCP Guidelines. The study protocol was also registered at clinicaltrials.gov and Australian New Zealand Clinical Trial Registry (ANZCTR) under the registration numbers NCT04164446 and ACTRN12619001786189, respectively.

Upon being selected as a volunteer, all potential candidates underwent a screening visit in January 2020 where the inclusion/exclusion criteria were detailed (as described in Section 2.3 Inclusion and Exclusion Criteria), and the study protocol and procedures were explained to them. The study coordinator answered any questions posed by the subjects, and their written informed consent was obtained. Thereafter, a medical examination, a general health questionnaire for the record of medical history, allergies, drug/supplements intake, and blood samples were utilized to confirm eligibility. Eligible volunteers were randomly assigned to four groups with 25 volunteers in each group; placebo/control group and groups who received either 250 mg of OPP, 1,000 mg of OPP, or 1,500 mg of OPP. Each of the volunteers needed to consume a total of four capsules per day for 60 days. Assignments into each study group remained concealed until the study was completed.

On day 1 of the trial, the selected volunteers attended the clinical trial ward (CTW) for a baseline blood sample and medical examination by physicians. During the trial, fasting blood samples were collected by a well-trained phlebotomist on day 1 (baseline), day 30, and day 60 using butterfly needles. A total of 20 ml of blood was withdrawn and transferred into tubes either with or without ethylenediamine tetra acetic acid (EDTA). The blood sample was then sent to a certified independent laboratory for further analysis tests such as hematology and liver and renal fasting blood profiles. The volunteers were required to come to the CTW daily for close monitoring in the first 2 weeks and once a week for the remaining trial period. For each visit, the volunteers were required to take all four capsules simultaneously in front of the study staff to ensure compliance and to observe the acceptability of swallowing the capsule. Vital signs (blood pressure and pulse rate) and weight measurements were also taken, and volunteers were given the option to see physicians to report any complaints or adverse events. Starting from week 3 onwards, they were given capsules in a bottle for a 1-week supply of capsules and were required to bring the bottle back the subsequent week for capsule counting and restock. Additionally, to ensure drug compliance with the capsules, subjects were also required to fill up the details of their daily intake of the capsules in a subject diary.

### 2.5 Sample Size and Randomization of Volunteers

The sample size was calculated according to Sakpal (2010), whereby a minimum of 19 volunteers per group was required for this study. However, considering subject dropouts during the trial, we recruited 25 volunteers per group. Therefore, in this randomized controlled trial, a sample size of 100 healthy volunteers consisting of 25 subjects in each arm with a two-sided 5% significance level and a power of 80% was used to study the safety of OPP supplementation with an acceptable dropout rate of up to 20%.

Stratified randomization was performed using statistical software, Stata 14.0, with an allocation ratio of 1:1:1:1. Participant randomization was stratified by age, gender, and total cholesterol. The distribution of age and total cholesterol was approximately normal and, therefore, volunteers were divided into two groups based on the mean value. In total, there were eight subgroups according to these three characteristics (for example, subgroup 1 included young men with low total cholesterol). The allocation sequence for all subgroups was generated by using a computer-generated list (seed number 123456).

### 2.6 Fasting Blood Parameters

There were three routine blood parameters that were measured in this trial; hematology, renal blood profile, and liver blood profile. All of the blood samples collected were sent to a third independent laboratory for analysis.

To measure hematology parameters, blood samples were collected into ethylenediamine tetra acetic acid (EDTA) tubes. Parameters that were measured in this profile were erythrocyte sedimentation rate (ESR), red blood cells (RBCs), hemoglobin (Hb), packed cell volume (PCV), mean corpuscular volume (MCV), mean corpuscular hemoglobin (MCH), mean corpuscular hemoglobin concentration (MCHC), platelet count, white blood cells (WBCs), neutrophils, lymphocytes, monocytes, eosinophils, and basophils.

As for renal and liver blood profiles, blood samples were collected into a plain tube, without any anti-coagulant. Measured renal parameters were serum urea, creatinine, calcium, phosphate, uric acid, sodium, potassium, and chloride. The liver parameters measured were total protein (TP), albumin, globulin, bilirubin, alkaline phosphatase (ALP), aspartate transaminase (AST), alanine transaminase (ALT), and gamma-glutamyl transferase (GGT).

### 2.7 Adverse Event Causality

All the adverse events either serious or minor were recorded in our case report form. For each event reported, the physician asked about the severity, duration, and frequency of event and they decided to temporarily or permanently stop the treatment. Causality and relation to OPP consumption were then determined. The Naranjo classification is a scale used in the majority of clinical trials to assess the causal link between an adverse event and a drug treatment. We used this scale to categorize any symptoms reported by our volunteers to determine whether the symptoms reported by them were caused by OPP consumption.

Appendix 1 shows a questionnaire that consists of 10 questions to determine the relationship of symptoms reported with OPP consumption. The total score is then used to categorize the probability that the adverse reaction is attributable to the drug: ≥9, definitely; 5–8, probably; 1–4, possibly; ≤0, doubtfully.

### 2.8 Statistical Analysis

Statistical analysis was conducted using Statistical Package for the Social Sciences (SPSS), version 22.0. Intention to treat analysis (ITT) was used to analyze primary outcomes. Missing variables were assigned using simple imputation. Mean imputation was performed for the missing variables at follow-up: AST, ALT, ASP (3.0% missing), and GGT (9% missing). Categorical data were presented as frequency and percentages. Continuous normally distributed data were presented as the mean and standard deviation (SD) while not normally distribute data were presented as the median and interquartile range (IQR).

Three-time points from baseline to 60 days were treated as the within-subjects factor (effect over time) and the differences between intervention groups (dosage amount) were treated as the between-subjects factor. A one-way ANOVA test was used to examine the difference in safety profile values among the four groups (between-subjects) at baseline, 30 days, 60 days, and the average (combination of all three values). Repeated measure ANOVA was used to compare the change between time periods within each group (within-subjects). A *p*-value of less than 0.05 was considered statistically significant.

## 3 Results

### 3.1 Volunteers’ Participation Throughout the Study

**FIGURE 1:**
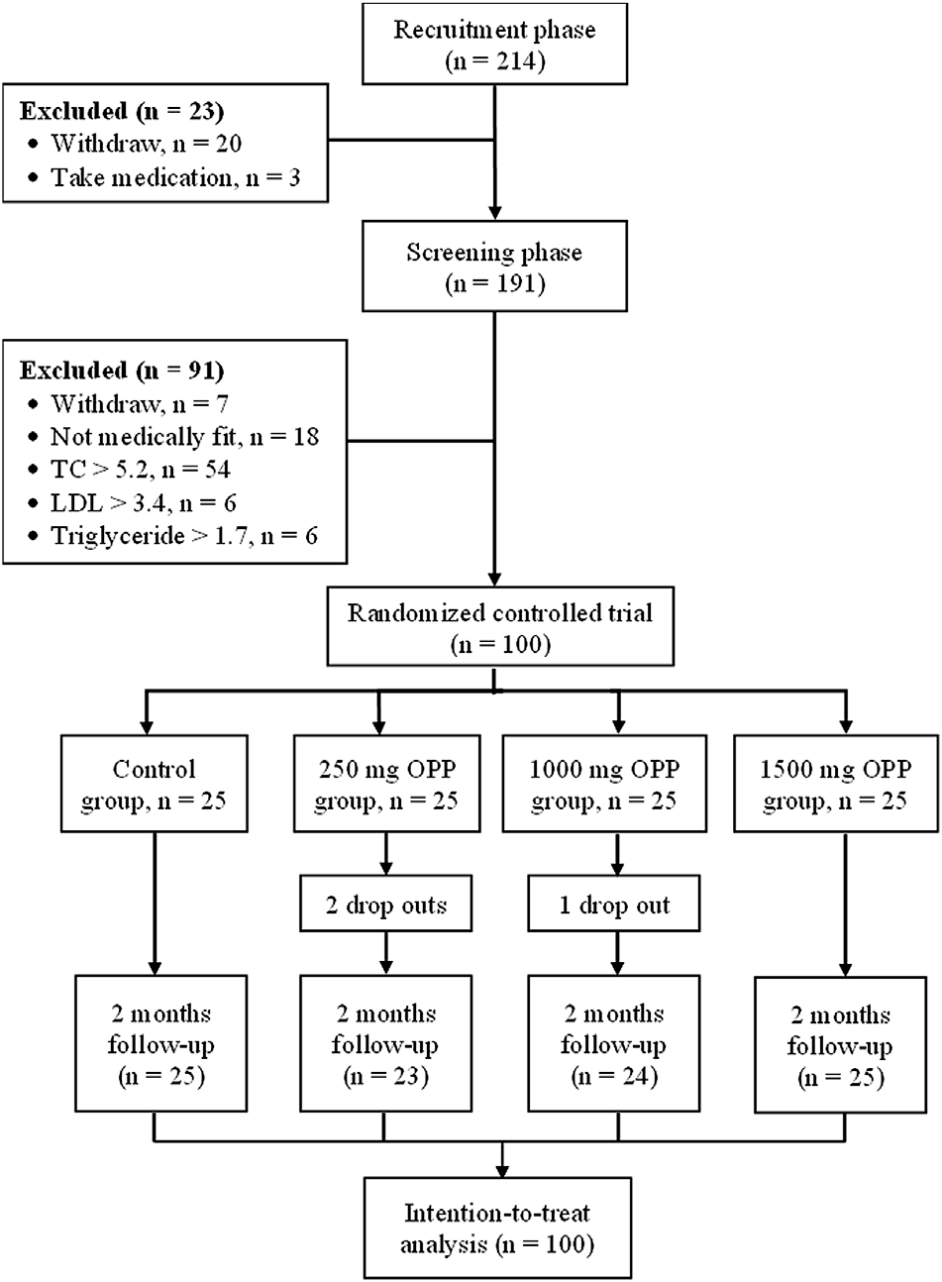
Flow diagram of an OPP supplementation clinical trial vs. placebo.

A total of 68 female and 32 male healthy volunteers met the entry requirements and were enrolled in the study. Overall, 97 of the participants completed the study, with three dropouts in the OPP groups. Figure 1 shows the flow diagram for the number of volunteers involved from the recruitment phase until the completion of the study.

### 3.2 Demographic and Baseline Clinical Characteristics

Basal anthropometric (BMI) and clinical characteristics are shown in Table 1. The baseline demographic characteristics were similar across all the groups. Data homogeneity between groups was achieved. No significant differences in the body mass index values or blood parameters were observed between the groups.

**TABLE 1:** Baseline characteristics of 100 healthy trial participants.

Parameters	Placebo (*n* = 25)	OPP			*p*
		250 mg (*n* = 25)	1,000 mg (*n* = 25)	1,500 mg (*n* = 25)	
Gender/sex					
Male (n)	8	7	8	9	0.947
Female (n)	17	18	17	16	
Age (years)	27.6 ± 5.6	29.2 ± 5.7	28.0 ± 6.2	27.9 ± 5.2	0.772
BMI (kgm^−2^)	23.62 ± 3.60	24.62 ± 5.44	23.69 ± 3.39	25.36 ± 5.20	0.472
Systolic (mmHg)	120.9 ± 10.0	127.2 ± 11.6	121.9 ± 12.0	125.7 ± 14.5	0.206
Diastolic (mmHg)	74.2 ± 8.6	79.6 ± 7.4	75.0 ± 9.8	78.8 ± 7.7	0.057
Total cholesterol (mmol/L)	4.66 ± 0.73	4.50 ± 0.63	4.47 ± 0.76	4.51 ± 0.65	0.772
LDL (mmol/L)	2.81 ± 0.64	2.72 ± 0.51	2.69 ± 0.68	2.74 ± 0.57	0.909
HDL (mmol/L)	1.46 ± 0.33	1.39 ± 0.29	1.43 ± 0.35	1.40 ± 0.24	0.883
Triglycerides (mmol/L)	0.86 ± 0.41	0.85 ± 0.49	0.76 ± 0.32	0.83 ± 0.29	0.760

Abbreviations: BMI, body mass index; LDL, low-density lipoprotein; HDL, high-density lipoprotein.

Data are tabulated as mean ± standard deviation or *n*.

### 3.3 Safety and Tolerance

All of the 97 volunteers reported that they were able to easily consume the capsules. Positive and negative feedback are tabulated accordingly to the four groups in Table 2 and Table 3, respectively. For the placebo group, two accounts of positive feedback were received: easy to defecate and increased appetite. The 250 mg OPP group received the highest positive feedback (*n* = 7), whereby easy to defecate (*n* = 2), feeling more energetic (*n* = 2), softer stool (*n* = 1), increased appetite (*n* = 1), and reduction in menstrual pain (*n* = 1). For the intermediate dose of OPP (1,000 mg), three positive responses were received referencing easy to defecate, increased appetite, and lose weight easily. Meanwhile, for the highest dose of OPP (1,500 mg), there were only two positive responses received: easy to defecate and increased appetite. As for the negative responses reported, all groups received two negative responses from the volunteers. For placebo groups, the volunteers complained about becoming hungry easily and losing appetite while for the 250 mg OPP group, each volunteer complained about intermittent chest discomfort and mild throat irritation. For the 1,000 mg OPP group, the complaints were of light-headedness and increased urinary frequency. Meanwhile, for the highest dose, the volunteers complained about bloating and delayed menses. All the adverse symptoms by the volunteers were categorized as “doubtful” according to the Naranjo classification of probability.

**TABLE 2:** Naranjo classification on positive effects of treatments from volunteers when questioned by a physician within 2 months of the trial.

Probability of Adverse Drug Reaction	Symptoms	Placebo (N)	OPP		
			250 mg (N)	1,000 mg (N)	1,500 mg (N)
Possible	Easy to defecate	1	2	1	1
	Softer stool	—	1	—	—
	Increase appetite	1	1	1	1
	Lose weight easily	—	—	1	—
	More energetic	—	2	—	—
Doubtful	Reduce menstrual pain	—	1	—	—
Total		2	7	3	2

Data are tabulated as (N).

**TABLE 3:** Naranjo classification on negative effects of treatments from volunteers when questioned by a physician within 2 months of the trial.

Probability of Adverse Drug Reaction	Symptoms	Placebo (N)	OPP		
			250 mg (N)	1,000 mg (N)	1,500 mg (N)
Doubtful	Get hungry easily	1	—	—	—
	Losing appetite	1	—	—	—
	Intermittent chest discomfort	—	1	—	—
	Mild throat irritation	—	1	—	—
	Light-headedness	—	—	1	—
	Increase urinary frequency	—	—	1	—
	Bloated	—	—	—	1
	Menses delay	—	—	—	1
Total		2	2	2	2

Data are tabulated as (N).

### 3.4 Changes in Body Weight

Weight changes in all volunteers in each intervention time point are shown in Table 4. All OPP-treated groups showed consistency in body weight changes from day 1 to day 60. Only the placebo group demonstrated a statistically significant reduction (*p* < 0.001) in body weight. However, clinically, this reduction was not significant as the reduction value was only 0.8 kg when compared to day 1 (baseline).

**TABLE 4:** Mean changes in volunteers’ body weight (kg) on day 1, day 30, and day 60.

	Day 1	Day 30	Day 60
Placebo	60.6 ± 12.5	60.4 ± 12.3	59.8 ± 12.4*
250 mg OPP	63.1 ± 17.4	63.5 ± 17.3	63.4 ± 16.9
1,000 mg OPP	60.9 ± 10.1	60.9 ± 10.4	60.1 ± 10.2
1,500 mg OPP	63.6 ± 12.7	63.8 ± 12.4	63.6 ± 12.6

Data are tabulated as mean ± standard deviation.

*Statistical differences compared to day 1 within the group (*p* < 0.05).

### 3.5 Hematology Parameters

Hematology parameters are displayed in Table 5. One-way ANOVA resulted in no significant difference between control groups and OPP-treated groups. Repeated measured ANOVA for OPP-treated groups showed statistically significant changes over time and all the parameters’ values were within the normal clinical range. In the 250 mg OPP group, red blood cell (RBC) (*p* = 0.033) and monocyte (*p* = 0.046) parameters showed a statistically significant increase following the intake of the OPP supplement. In the 1,000 mg group, parameters that statistically significant increased were RBCs (*p* = 0.001), hemoglobin (Hb) (*p* = 0.005), and packed cell volume (PCV) (*p* = 0.019). The highest dose of OPP had numerous parameters that significantly increased: ESR (*p* = 0.036), RBCs (*p* = 0.003), Hb (*p* = 0.004), PCV (*p* = 0.001), and neutrophils (*p* = 0.044).

**TABLE 5:** Hematology parameter values at baseline, day 30, and day 60.

	Control	250 mg	1,000 mg	1,500 mg
ESR (mm/hr)				
Day 1	10.44 ± 12.42	11.67 ± 10.94	13.20 ± 13.80	10.16 ± 10.9
Day 30	11.20 ± 9.40	13.96 ± 9.46	11.08 ± 9.95	15.36 ± 13.8^a^
Day 60	13.17 ± 10.95	12.09 ± 9.52	12.09 ± 9.52	13.54 ± 13.30
RBCs (10^12^/L)				
Day 1	4.84 ± 0.65	4.91 ± 0.60	4.83 ± 0.55	4.96 ± 0.56
Day 30	4.84 ± 0.63	4.96 ± 0.50	4.86 ± 0.50	4.95 ± 0.55
Day 60	4.91 ± 0.57	5.06 ± 0.56^a^	5.00 ± 0.59^c^	5.13 ± 0.56^b^
Hb (g/dl)				
Day 1	13.60 ± 1.44	13.32 ± 1.67	13.05 ± 1.62	13.08 ± 1.81
Day 30	13.60 ± 1.36	13.42 ± 1.46	13.15 ± 1.74	13.06 ± 1.64
Day 60	13.75 ± 1.41	13.62 ± 1.64	13.43 ± 1.84^b^	13.56 ± 1.69^b^
PCV(%)				
Day 1	41.2 ± 4.0	41.0 ± 4.9	39.8 ± 4.2	40.7 ± 4.2
Day 30	41.4 ± 3.8	41.5 ± 4.2	41.4 ± 7.1	41.0 ± 4.0
Day 60	41.9 ± 3.9	42.1 ± 4.5	40.8 ± 4.7^b^	42.0 ± 4.2^c^
MCV (fL)				
Day 1	85.4 ± 7.4	83.5 ± 5.5	82.8 ± 7.2	82.3 ± 7.8
Day 30	86.2 ± 5.9	83.9 ± 5.7	81.1 ± 14.3	83.2 ± 7.8
Day 60	85.7 ± 6.7	83.3 ± 5.3	82.3 ± 8.0	82.4 ± 7.1
MCH (pg)				
Day 1	28.3 ± 2.5	27.3 ± 2.1	27.2 ± 3.0	26.6 ± 3.5
Day 30	28.4 ± 2.4	27.2 ± 2.0	27.6 ± 3.0	26.5 ± 3.3
Day 60	28.2 ± 2.5	27.1 ± 2.2	27.0 ± 3.4	26.7 ± 3.1
MCHC (10^9^/L)				
Day 1	33.0 ± 1.3	32.6 ± 1.0	32.8 ± 1.6	32.2 ± 1.6
Day 30	32.8 ± 1.3	32.4 ± 1.2	32.7 ± 1.6	32.0 ± 1.4
Day 60	32.8 ± 1.2	32.4 ± 1.8	33.0 ± 1.5	32.4 ± 1.5
Platelet (10^9^/L)				
Day 1	299.6 ± 59.6	302.9 ± 63.5	304.5 ± 84.5	325.3 ± 93.8
Day 30	306.7 ± 53.8	300.4 ± 56.1	319.8 ± 74.4	336.3 ± 93.5
Day 60	287.4 ± 45.9	291.0 ± 61.3	316.2 ± 76.7	321.5 ± 93.1
WBCs (10^9^/L)				
Day 1	6.81 ± 1.47	7.36 ± 1.87	7.04 ± 1.40	6.98 ± 1.51
Day 30	6.59 ± 1.37	7.33 ± 2.18	6.71 ± 1.13	6.68 ± 1.65
Day 60	6.69 ± 1.56	6.91 ± 1.86	6.98 ± 1.50	6.68 ± 1.34
Neutrophil (%)				
Day 1	55.3 ± 9.8	51.9 ± 6.6	53.9 ± 9.2	51.9 ± 9.5
Day 30	54.6 ± 8.3	53.3 ± 5.9	53.7 ± 8.9	54.5 ± 7.9^b^
Day 60	52.7 ± 8.3	53.0 ± 6.9	53.8 ± 6.7	52.6 ± 9.4
Lymphocytes (%)				
Day 1	33.8 ± 8.1	37.4 ± 6.8	35.1 ± 8.8	36.2 ± 8.8
Day 30	34.3 ± 7.1	35.9 ± 5.9	35.3 ± 6.9	34.1 ± 7.5^b^
Day 60	35.4 ± 7.1	37.1 ± 7.0	35.5 ± 5.5	36.4 ± 9.2
Monocytes (%)				
Day 1	6.46 ± 1.49	6.86 ± 1.94	7.26 ± 1.55	6.96 ± 1.43
Day 30	6.68 ± 1.32	7.44 ± 1.48^b^	7.45 ± 1.73	7.10 ± 1.60
Day 60	7.02 ± 1.67	6.53 ± 1.19	7.20 ± 1.50	6.61 ± 1.22
Eosinophils (%)				
Day 1	3.77 ± 3.28	3.22 ± 1.82	2.95 ± 2.35	4.23 ± 2.42
Day 30	3.80 ± 2.95	2.65 ± 1.14	2.80 ± 2.27	3.62 ± 1.89
Day 60	4.12 ± 4.12	2.79 ± 1.29	2.99 ± 1.70	3.87 ± 2.26
Basophil (%)				
Day 1	0.68 ± 0.41	0.62 ± 0.30	0.78 ± 0.35	0.72 ± 0.37
Day 30	0.62 ± 0.39	0.65 ± 0.31	0.76 ± 0.32	0.72 ± 0.42
Day 60	0.69 ± 0.34	0.66 ± 0.36	0.56 ± 0.33^a^	0.49 ± 0.32

Abbreviations: ESR, erythrocyte sedimentation rate; RBCs, red blood cells; Hb, hemoglobin; PCV, packed cell volume; MCV, mean corpuscular volume; MCH, mean corpuscular hemoglobin; MCHC, mean corpuscular hemoglobin concentration; WBCs, white blood cells.

a Significantly different compared to day 1 within the group (*p* < 0.05).

b Significantly different compared to day 1 within the group (*p* < 0.010).

c Significantly different compared to day 1 within the group (*p* = 0.001).

Data are presented as mean ± standard deviation.

### 3.6 Renal Function Test

Changes in the RFT parameters are displayed in Table 6. One-way ANOVA resulted in no significant difference between control and OPP-treated groups. Repeated measure ANOVA demonstrated changes of the parameters in all groups throughout the 60 days and all measured values were within the normal range. In the placebo group, creatinine, calcium, and sodium were statistically increased. In the 250 mg OPP group, calcium, sodium, and potassium were statistically increased. In the 1,000 mg OPP group, there was a statistically significant elevation of calcium, sodium, potassium, and chloride while the 1,500 mg OPP group had a significant increase in creatinine, calcium, phosphate, sodium potassium, and chloride. All OPP-treated groups showed a statistically significant increase in calcium and potassium.

**TABLE 6:** Serum renal function test at baseline, day 30, and day 60.

	Control	250 mg	1,000 mg	1,500 mg
Urea (mmol/L)				
Day 1	4.16 ± 0.92	4.23 ± 1.08	3.76 ± 0.89	4.01 ± 0.90
Day 30	4.07 ± 0.98	4.38 ± 1.08	3.94 ± 0.76	4.08 ± 1.00
Day 60	4.05 ± 0.84	3.99 ± 0.97	3.86 ± 0.87	4.07 ± 1.06
Creatinine (mmol/L)				
Day 1	64.9 ± 13.3	63.0 ± 16.7	62.1 ± 16.5	68.5 ± 16.3
Day 30	66.6 ± 13.2	65.6 ± 13.4	63.5 ± 15.7	71.1 ± 16.5^a^
Day 60	67.3 ± 14.3^a^	62.3 ± 19.1	63.0 ± 14.4	69.8 ± 16.8
Calcium (mmol/L)				
Day 1	2.28 ± 0.08	2.26 ± 0.09	2.29 ± 0.07	2.31 ± 0.07
Day 30	2.34 ± 0.08^b^	2.34 ± 0.09^c^	2.36 ± 0.11^c^	2.38 ± 0.10^c^
Day 60	2.30 ± 0.08	2.32 ± 0.10	2.33 ± 0.11	2.35 ± 0.11
Phosphate (mmol/L)				
Day 1	1.23 ± 0.15	1.19 ± 0.16	1.25 ± 0.16	1.20 ± 0.16
Day 30	1.25 ± 0.18	1.27 ± 0.17	1.28 ± 0.14	1.27 ± 0.16^a^
Day 60	1.24 ± 0.16	1.33 ± 0.51	1.29 ± 0.15	1.25 ± 0.17
Uric acid (mmol/L)				
Day 1	0.35 ± 0.09	0.32 ± 0.08	0.32 ± 0.10	0.33 ± 0.08
Day 30	0.34 ± 0.08	0.32 ± 0.07	0.31 ± 0.08	0.34 ± 0.07
Day 60	0.34 ± 0.09	0.31 ± 0.09	0.32 ± 0.08	0.32 ± 0.07
Sodium (mmol/L)				
Day 1	140.3 ± 1.7	139.8 ± 1.3	139.5 ± 1.7	139.9 ± 2.1
Day 30	143.4 ± 2.0^c^	142.1 ± 1.9^a^	142.7 ± 2.4^c^	143.1 ± 2.0^c^
Day 60	139.6 ± 2.0	138.7 ± 1.6	138.8 ± 1.9	139.2 ± 2.0
Potassium (mmol/L)				
Day 1	3.97 ± 0.37	4.12 ± 0.56	4.03 ± 0.33	4.08 ± 0.37
Day 30	4.41 ± 0.59^c^	4.42 ± 0.48^a^	4.46 ± 0.35	4.41 ± 0.37
Day 60	4.35 ± 0.58	4.44 ± 0.56	4.51 ± 0.80^b^	4.71 ± 0.56^p^
Chloride (mmol/L)				
Day 1	103.2 ± 1.5	103.6 ± 2.2	102.9 ± 1.7	103.8 ± 1.7
Day 30	104.9 ± 1.2^b^	104.3 ± 2.0	104.4 ± 2.0^c^	105.0 ± 1.6^c^
Day 60	103.8 ± 1.9	102.5 ± 1.9^a^	103.6 ± 2.3	103.8 ± 1.9

a Significantly different compared to day 1 within the group (*p* < 0.05).

b Significantly different compared to day 1 within the group (*p* < 0.010).

c Significantly different compared to day 1 within the group (*p* = 0.001).

Data are presented as mean ± standard deviation.

### 3.7 Liver Function Test

Changes in the liver function test parameters are displayed in Table 7. Control and OPP-treated groups did not have any significant differences when analyzed using one-way ANOVA, while repeated measure ANOVA analysis resulted in a significant increase in total protein (TP) and globulin in all groups. In addition, the control group had a significant decrease in alkaline phosphatase (ALP) (*p* = 0.009) from day 1 to day 60, the low dose (250 mg) group had a significant decrease in alanine aminotransferase (ALT) (*p* = 0.026), the 1,000 mg-treated group had a significant increase in bilirubin (*p* = 0.034), and the highest dose (1,500 mg)-treated group had a significant increase in ALT (*p* = 0.013) and gamma-glutamyl transferase (GGT) (*p* = 0.001) after 60 days. Despite these changes, the actual values were within the normal range.

**TABLE 7:** Serum liver function test at baseline, day 30, and day 60.

	Control	250 mg	1,000 mg	1,500 mg
Total protein (g/L)				
Day 1	71.9 ± 3.9	72.2 ± 3.0	71.9 ± 4.3	72.8 ± 2.8
Day 30	73.2 ± 4.2	74.3 ± 2.8	71.5 ± 15.0	74.8 ± 3.6
Day 60	73.8 ± 4.4	75.0 ± 4.3^a^	74.5 ± 4.0^b^	75.6 ± 4.3^b^
Albumin (g/L)				
Day 1	45.7 ± 2.2	46.0 ± 2.4	45.5 ± 2.5	46.3 ± 2.3
Day 30	45.7 ± 2.1	46.7 ± 2.0	47.2 ± 5.4	46.9 ± 2.1
Day 60	45.9 ± 2.5	46.2 ± 2.6	45.3 ± 3.6	46.2 ± 3.2
Globulin (g/L)				
Day 1	26.2 ± 3.3	26.2 ± 3.1	26.4 ± 3.3	26.4 ± 2.6
Day 30	27.5 ± 3.3	27.5 ± 2.9	28.8 ± 4.9	27.9 ± 3.1
Day 60	27.9 ± 3.6^b^	28.8 ± 3.8^a^	31.2 ± 9.8^c^	29.3 ± 4.2^b^
Bilirubin (μmol/L)				
Day 1	11.44 ± 3.61	11.40 ± 4.50	11.12 ± 4.07	11.52 ± 5.26
Day 30	12.04 ± 5.05	12.67 ± 6.01	12.84 ± 5.63^c^	10.72 ± 4.16
Day 60	13.12 ± 6.67	12.78 ± 5.69	11.92 ± 5.50	10.40 ± 4.53
ALP (IU/L)				
Day 1	59.6 ± 16.6	67.0 ± 17.8	60.9 ± 17.3	69.2 ± 12.2
Day 30	59.6 ± 15.6	65.8 ± 13.8	62.2 ± 18.4	70.3 ± 10.9
Day 60	55.9 ± 15.3^b^	66.4 ± 16.4	59.2 ± 14.2	67.9 ± 10.3
AST (IU/L)				
Day 1	20.7 ± 10.4	21.0 ± 8.8	18.7 ± 3.9	18.8 ± 4.2
Day 30	18.8 ± 3.7	20.1 ± 5.3	19.0 ± 3.8	20.0 ± 4.6
Day 60	18.7 ± 4.2	21.0 ± 7.0	19.4 ± 3.6	19.8 ± 4.4
ALT (IU/L)				
Day 1	19.9 ± 14.5	21.2 ± 16.2	20.4 ± 17.9	18.2 ± 7.1
Day 30	18.2 ± 10.5	17.3 ± 10.4^c^	15.6 ± 6.3	18.8 ± 8.8
Day 60	18.4 ± 12.5	21.8 ± 14.9	17.7 ± 10.3	22.0 ± 10.1^c^
GGT (IU/L)				
Day 1	21.0 ± 24.6	22.0 ± 17.7	17.6 ± 13.1	17.4 ± 7.6
Day 30	20.0 ± 20.6	19.0 ± 10.5	18.2 ± 14.0	19.0 ± 10.7
Day 60	19.0 ± 18.0	23.9 ± 16.9	19.7 ± 12.6	21.3 ± 7.2^c^

Abbreviations: ALP, alkaline phosphatase; AST, aspartate transaminase; ALT, alanine transaminase; GGT, gamma-glutamyl transferase.

a Significantly different compared to day 1 within the group (*p* = 0.001).

b Significantly different compared to day 1 within the group (*p* < 0.010).

c Significantly different compared to day 1 within the group (*p* < 0.05).

Data are presented as mean ± standard deviation.

## 4 Discussion

The primary endpoint of this trial is to assess the tolerability and safety of OPP supplementation. There was minimal or no reports of serious adverse events (SAEs), no drastic changes in weight measurement, and normal blood measurements based on hematology, liver, and renal profiles. All OPP-treated groups had a statistically significant increase RBCs, Hb, and PCV from day 1 to day 60 although the changes were not clinically significant and were well within the normal range. Although not clinically significant, the increased pattern in certain blood parameters of all OPP-treated groups could have been associated with erythropoiesis. Erythropoiesis is the production process of RBCs and this process is stimulated by the erythropoietin hormone, as a response of low oxygen content. The increased rate of erythropoiesis will increase the volume of RBCs, resulting in increased volume of Hb (iron-containing oxygen-transport metalloprotein in the red blood cells) and PCV (percentage of red blood cells in circulating blood) (Dzierzak and Philipsen, 2013). This finding is supported by Essien et al. (2014) where similar increases in RBCs, Hb, and PCV in rats given with palm oil feed for 90 days were observed. OPP has been shown to upregulate erythroid derived-nuclear factor 2-like 1 (NFE2L1) (Leow et al., 2011), which is associated with cellular homeostasis, normal organ development, and growth during life processes (Zhang and Xiang, 2016). In an experiment using NFE2L1 knockout mice, impairment of fetal liver erythropoiesis was observed, which indicates the important role of NFE2L1 in EPO (Chan et al., 1998). Therefore, the stimulation of RBC production by OPP might be associated with the ability to upregulate the NFE2L1 gene.

The renal function test (RFT) and liver function test (LFT) are the main safety parameters used to determine the safety of investigated products in our clinical trial. Any significant changes resulting in an abnormal result especially serum AST, ALT, creatinine, and urea indicate the tested product are harmful to the consumer. Serum AST and ALT are vital enzymes present in the liver, and abnormal high levels indicate liver damage while increased serum creatinine and urea indicate renal failure (Goorden et al., 2013; Salazar, 2014). In this trial, the OPP supplement was considered safe for consumption by human subjects at the range of 250–1,500 mg indicated by no significant differences in the RFT and LFT values between control and OPP-treated groups. Our study was in parallel with a previous single-blinded clinical trial performed by Fairus et al. (2018), whereby OPP was given up to 9,000 mg per day in fluid form (300 ml per day for 60 days) to human subjects. They reported that the subjects did not experience any major adverse events (AEs). Therefore, our study demonstrated that the encapsulated form of OPP was safe and could be consumed at doses up to 1,500 mg a day. The lowest and intermediate dose of OPP (250 and 1,000 mg) were selected based on the previous animal study by Syarifah-Noratiqah. (2021). Meanwhile, the highest dose of OPP (1,500 mg) was included to determine the upper tolerability of OPP based on the safety profile conducted by Lynch et al. (2017). All the animal data from the previous studies were extrapolated to human doses (Nair and Jacob, 2016).

In our clinical trial, volunteers in all groups were required to take four OPP capsules simultaneously every day for 60 days. Since our aim was to simulate real-life supplementation intake, we did not set any conditions for the capsules to be taken. Volunteers were free to choose when to take the capsule, either during the day or night, before or after a meal. However, volunteers were not allowed to take other antioxidant supplements throughout the trial. This restriction was vital to ensure the results of the trial were due to OPP itself (OPP is an antioxidant agent), not the combination of other antioxidant supplements. There were no reports of allergies to OPP supplementation and there were no serious adverse events (SAEs) reported. Although few parameters in hematology and biochemistry blood results showed statistically significant changes, all the values for all volunteers remained within the normal range according to the standard clinical safety references. All these results indicate that dosage of OPP supplementation in capsule form is safe up to 1,500 mg.

Throughout the trial, there were positive responses from volunteers when they were consulted by the physician, with the majority of reports from the volunteers in the 250 mg OPP group. Positive feedback reported included soft stool and easy to defecate, increase in appetite without gaining weight, and satiety for a long period after a meal. These positive responses are similar to anti-obesity agents as described by Rodgers et al. (2012). The potential use of phenolics components as anti-obesity agents has been actively investigated (Amaya-Cruz et al., 2019; El-shiekh et al., 2019; Hsu and Yen, 2008; Sergent et al., 2012; Yen et al., 2020). Plant polyphenols are capable of preventing obesity by lowering food intake, reducing lipogenesis, increasing lipolysis, stimulating fatty acids (FA) β-oxidation, and suppressing oxidative stress (Wang et al., 2014). The main phenolic components of OPP are caffeoyl shikimic acid which has a vital role in anti-obesity mechanisms as observed in the *Aloe ferox* leaf powder (Mokhele et al., 2020), *Hibiscus sabdariffa* extract (Diez-Echave et al., 2020; Yen et al., 2020), and yerba mate (Burris et al., 2012). The possible explanation on volunteers feeling satiety for longer might be due to the retarding action of gastrointestinal carbohydrate hydrolyzing enzymes which delay carbohydrate breakdown, thus, prolong its digestion time and decrease the rate of glucose absorption (Olaokun et al., 2013). OPP was also noted to have beneficial effects on the large bowel such as increasing the rate of distal colonic contractility and motility (Patten et al., 2015), increasing the bulking of the stool, promoting the production of short chain fatty acids (SCFAs), altering the certain gut microbiome by increasing their cecal digesta (Conlon et al., 2020), and metabolizing these compounds into further bioactive molecules that produce health benefits (Leow et al., 2021). All these effects of OPP might explain some of subject’s feedback on soft stools and easier defecation. In term of weight changes, there were no statistically significant weight changes from day 1 to day 60 in the OPP-tested groups despite the volunteers’ feedback of weight loss when interviewed by the physician. Only the control group showed a statistically significant reduction in body weight from day 30 (60.4 ± 12.3) to day 60 (59.8 ± 12.4) although clinically, the changes were very minor (less than 2%) and the reduction pattern did not cause any health concerns. According to Ebbert et al. (2014), a reduction of 5% body weight is considered a clinically significant reduction. Moreover, no significant weight changes were observed in the treated groups.

In terms of adverse effects, only 6 out of the 75 volunteers in the OPP-treated groups complained of adverse effects such as intermittent chest discomfort, mild throat irritation, lightheadedness, increased urinary frequency, feeling nausea, and menses delay. All these symptoms appeared temporarily and occurred only once in the volunteers. The Naranjo scoring method was used to specify causal links between symptoms and treatments given (Naranjo et al., 1981). All eight reported adverse effects fall under the “doubtful” category after Naranjo assessments were completed (Appendix 1).

There were a few minor adverse events (AEs) and no serious adverse events (SAEs) were reported throughout the trial. The majority of subjects claimed that their health had improved when taking the OPP supplement. Clinical biochemistry (renal and liver profile tests) and hematology tests for all subjects showed no abnormality indicating daily oral consumption of the OPP capsule was safe. In conclusion, OPP supplementation in the capsule form is safe up to 1,500 mg (Syarifah-Noratiqah, 2021).

## Data Availability

The raw data supporting the conclusion of this article will be made available by the authors, without undue reservation.

## Appendix

Adverse drug reaction scale according to Naranjo classification. To evaluate the adverse drug reaction, the questionnaire was finalized by entering a score in the last column for each question and totaling the individual scores. The total score was then used to categorize the probability that the adverse reaction was attributable to the drug: ≥9, definitely; 5–8, probably; 1–4, possibly; ≤0, doubtfully.

**Table:**

No.	Question	Yes	No	Do Not Know	Score
1	Are there previous conclusive reports on this reaction?	+1	0	0	
2	Did the adverse event appear after the suspected drug was administered?	+2	−1	0	
3	Did the adverse event improve when the drug was discontinued or a specific antagonist was administered?	+1	0	0	
4	Did the adverse event reappear when the drug was readministered?	+2	−1	0	
5	Are there alternative causes that could on their own have caused the reaction?	−1	+2	0	
6	Did the reaction reappear when a placebo was given?^a^	−1	+1	0	
7	Was the drug detected in blood or other fluids in concentrations known to be toxic?	+1	0	0	
8	Was the reaction more severe when the dose was increased or less severe when the dose was decreased?^a^	+1	0	0	
9	Did the patient have a similar reaction to the same or similar drugs in any previous exposure?	+1	0	0	
10	Was the adverse event confirmed by any objective evidence?	+1	0	0	
		Total score			

^a^Answers to questions 6–8 were unknown since we did not perform the procedure.
